# Mesenchymal Stem Cells Ameliorate Hyperglycemia in Type I Diabetic Developing Male Rats

**DOI:** 10.1155/2022/7556278

**Published:** 2022-04-13

**Authors:** Hany N. Yousef, Samia M. Sakr, Sahar A. Sabry

**Affiliations:** Department of Biological and Geological Sciences, Faculty of Education, Ain Shams University, Cairo 11566, Egypt

## Abstract

One of the most promising treatments for diabetes mellitus (DM) is stem cell therapy. This study is aimed at elucidating the antidiabetic effect of mesenchymal stem cells (MSCs) on streptozotocin- (STZ-) induced DM in developing male rats. Twenty-four male albino rats (4 weeks old) were divided into control, diabetic, diabetic+MSCs1 (received MSCs one week after STZ treatment), and diabetic+MSCs2 (received MSCs 4 weeks after STZ treatment). Diabetic rats showed marked impairment (*p* < 0.05) in serum levels of glucose, insulin, C-peptide, glycosylated hemoglobin (HbA1c), malondialdehyde (MDA), total antioxidant status (TAS), and total oxidant status (TOS) in addition to disruption of the calculated values of homeostatic model assessment of insulin resistance (HOMA-IR), pancreatic *β* cell function (HOMA-*β*), and oxidative stress index (OSI). These biochemical alterations were confirmed by the histopathological and ultrastructural assessments which showed marked destructive effect on pancreatic islet cells. MSC therapy in an early stage reversed most of the biochemical, histological, and ultrastructural alterations in the STZ-induced diabetic model and restored the normal cellular population of most acinar cells and islet of Langerhans. These results indicate that MSC therapy of STZ-induced diabetic developing rats during an early stage has the capacity of *β* cell restoration and the control of blood glycemic homeostasis.

## 1. Introduction

Diabetes mellitus (DM) is among the most important endocrine disorders and public healthcare problems worldwide characterized by hyperglycemia as its hallmark feature. This disease results either from a loss of insulin-producing pancreatic *β* cells which is referred to as type I diabetes mellitus (T1DM) or through the mislaying of hormone responsiveness in its target tissues like adipose tissues and muscles which is known as type II diabetes mellitus (T2DM) [1]. The stubborn blood sugar concentration resulting as a consequence of diabetes generates free radicals causing oxidative stress that plays a substantial role in the pathophysiology of diabetes. Patients with DM have a high risk for many serious health disorders and may be subject to the irreversible injury, malfunction, and defect of many organs, including the skin, kidney, eye, nerves, and heart [2].

Due to the adverse effects caused by hyperglycemia on the different body organs, much research adopted various trails to overcome its pathological impacts. Patients with T1DM depend upon daily insulin injections; however, exogenous uptake of insulin can never be as accurate and dynamic like insulin secretion from endogenous *β* cells and therefore can only partially diminish the risk of the development of the disease complications [3].

So far, the trials to develop efficient immunosuppressive remediation to restrain *β* cell loss before DM onset had limited success [4]. Consequently, the reestablishment of endogenous insulin secretion represents a very important goal to maintain the blood sugar concentration within its normal range as well as to lessen or avoid complications of hyperglycemia and the patient's need for the self-management of blood glucose by exogenous insulin administration. Recent progress in regenerative medicine has capitalized on employing stem cells for the repair and regeneration of different cells and tissues. In this context, the transplantation of insulin-secreting cells obtained from human embryonic stem cells (hESCs) has been suggested as a therapeutic regimen for DM [5]. Nonetheless, the ethical quandary surrounding the destruction of a human embryo was and continues to be a major impediment to the development of therapeutic medicines based on human embryonic stem cells (hESCs) [6]. Individual attitudes are so firmly embedded in basic moral values that constructing a definite policy that everyone can agree on appears implausible. This ethical quandary is reflected in many pieces of laws governing hESC research across the world [7].

Mesenchymal stem cells (MSCs) have emerged as hopeful candidates to cure many diseases due to their distinctive characteristic properties like self-renewal, immune-suppressive potential, and capability to transdifferentiate. They are easily obtained from varied tissue sources, like umbilical cord, bone marrow (BM), and adipose tissues, with the minimal risk of rejection, and many previous studies have provided evidences of their valuable roles. MSCs are employed in dermal repair and regeneration in severe burning and diabetic wounds [8], besides their role in restoring and regenerating the *β* cells of the pancreas [9, 10]. To the best of our knowledge, very few trials have been conducted on the beneficial role of MSCs in ameliorating T1DM in developing mammals. Therefore, the aim of the current work was to elucidate the potential of MSC infusion in ameliorating hyperglycemia in streptozotocin- (STZ-) induced diabetic developing male rats, monitored by biochemical, histological, and ultrastructural approaches.

## 2. Materials and Methods

### 2.1. Experimental Animals

Twenty-four male developing albino rats (4 weeks old) with body weights ranging from 60 to 80 g and at the age of one month were obtained from the Egyptian Organization for Biological Products and Vaccines, Giza, Egypt. The animals were housed in clear plastic cages (2 animals/cage) with wood chips as bedding in a room with controlled conditions (temperature range of 25 ± 2°C, relative humidity of 55 ± 5%, and a 12 h light/dark cycle). The animals were fed ad libitum with a standard diet and acclimatized in the laboratory for one week (from postnatal day 28 to postnatal day 35) prior to experimentation. The experiments were performed parallel to the ethical standards and according to the guide approved by the Local Institutional Animal Ethics Committee of Ain Shams University.

### 2.2. Induction of Experimental Diabetes

Rats fasted for 12 h have been rendered diabetic by a single intraperitoneal (IP) injection of freshly prepared STZ (Sigma-Aldrich, St. Louis, MO, USA) at a dose of 45 mg/kg of body weight dissolved in ice-cold saline (pH = 4.5) in a volume of 1 ml/kg of body weight [11]. To avoid hypoglycemia and mortality, rats were permitted to drink 5% glucose solution ad libitum overnight after STZ injection. Blood samples were taken from the tail vein 72 h after STZ administration, and the fasting blood glucose (FBG) concentration was determined by means of one touch ultra-glucometer and compatible blood glucose strips. Rats exhibiting FBG ≥ 250 mg/dl were considered diabetic and were selected for the experiments. Control rats were injected with normal saline solution parallel to the treated groups throughout the course of the study.

### 2.3. Preparation and Isolation of Bone Marrow- (BM-) Derived Mesenchymal Stem Cells (MSCs)

Bone marrow was extracted from 10 adult male white albino rats' tibiae and femurs by flushing them with Dulbecco's modified Eagle's medium (DMEM) combined with 10% foetal bovine serum. A density gradient was used to separate nucleated cells, which were then resuspended in a complete culture medium supplemented with 1% penicillin–streptomycin. As a primary culture or upon formation of large colonies, cells were incubated at 37°C in 5% humidified CO_2_ for 12–14 days. As large colonies were formed (80–90% confluence), the cultures were washed twice with phosphate buffer saline (pH 7) and trypsinized for 5 minutes at 37°C with 0.25% trypsin in 1 mM EDTA. Cells were resuspended in serum-augmented medium after centrifugation and incubated in 50 cm^2^ culture flasks (Falcon). The resulting cultures were considered as first-passage cultures according to Alhadlaq and Mao [12]. MSCs are defined by their fusiform outline and adhesiveness [13].

Cells were resuspended in a wash buffer (BD Biosciences, Germany) after a short centrifugation. For 45 minutes at room temperature, 300 ml of cell suspension was incubated with antibodies against CD29, CD105, CD34, and CD90 conjugated with allophycocyanin (APC), cyanine 5 (CY5), phycoerythrin (PE), and fluorescein isothiocyanate (FITC) dyes, respectively. A FACSCalibur (BD Biosciences, Germany) was used for flow cytometry, and Cell Quest software was used for analysis.

### 2.4. Experimental Design

The rats were divided into four groups (6 animals each) as follows.

Group I (control group): healthy rats received normal saline solution parallel to the treated groups throughout the course of the study.

Group II (diabetic group): STZ-induced diabetic rats.

Group III (diabetic+MSCs1 group): a single dose of MSCs (0.5 ml containing 2 × 10^6^ cells/rat) was injected via the tail vein [14] in rats with STZ-induced diabetes. This dose was conducted during the early phase (performed one week after induction of diabetes, i.e., one-week diabetic developing rats).

Group IV (diabetic+MSCs2 group): a single dose of MSCs (0.5 ml containing 2 × 10^6^ cells/rat) was injected via the tail vein (as in group III) into diabetic rats during the late phase (4 weeks after induction of diabetes, i.e., 4-week diabetic developing rats).

### 2.5. Collection of Sera and Tissue Samples

After 7 days of MSC injection, animals were fasted overnight and afterwards anesthetized under light ether anesthesia. Blood samples were gathered by cardiac puncture and then centrifuged at 1500 × *g* for 10 min at 4°C to get sera that were immediately stored at -80°C until use. Pancreatic tissue samples were also taken from rats of all groups for histological and ultrastructural studies.

### 2.6. Serum Biochemical Analysis

#### 2.6.1. Glucose, Insulin, C-Peptide, and Glycosylated Hemoglobin

Serum glucose concentrations were assayed following glucose oxidase method [15], whereas rat-specific Enzyme-Linked ImmunoSorbent Assay kits were used to quantify serum levels of insulin (ELISA, Crystal Chem, Elk Grove Village, IL, USA) and C-peptide (ELISA, DRG Instruments, GmbH, Marburg, Germany). The levels of glycosylated hemoglobin (HbA1c) were estimated in sera according to the method previously described by Nayak and Pattabiraman [16].

#### 2.6.2. Homeostatic Model Assessment

Homeostatic model assessment of insulin resistance (HOMA-IR) and pancreatic *β* cell function (HOMA-*β*) were calculated using fasting serum insulin and fasting serum glucose concentrations according to the following equations [17]: HOMA‐IR = fasting serum insulin (*μ*IU/ml) × fasting serum glucose (mmol/l)/22.5; HOMA‐*β* = fasting serum insulin (*μ*IU/ml) × 20/fasting serum glucose (mmol/l) − 3.5.

#### 2.6.3. Oxidative Stress Parameters

Lipid peroxidation (LP) was measured following the method of Draper and Hadley [18]. This method depends on the ability of malondialdehyde (MDA), a secondary product of lipid peroxidation, to react with thiobarbituric acid reactive substances (TBARS). The absorbance of the resulting colored product was measured spectrophotometrically at 532 nm.

Serum total antioxidant status (TAS) levels were determined following Erel's method [19], which is based on bleaching the green-bluish color of 2,2′-azino-bis (3-ethylbenz-thiazoline 6-sulfonic acid) (ABTS) radical cation by sample antioxidants. The assay was calibrated using a stable antioxidant standard solution known as Trolox equivalent (Trolox Eq), and the results were expressed in mmol Trolox Eq/l.

Serum total oxidant status (TOS) levels were measured according to the method described by Erel [20]. This method is based on oxidation of ferrous ion–chelator complex into ferric ion by the various oxidants in the sample under acidic conditions. The assay was calibrated using hydrogen peroxide, and the results were expressed in *μ*mol H_2_O_2_ Eq/l.

Values of oxidative stress index (OSI) were calculated using the following formula: OSI (arbitrary unit) = TOS (*μ*mol H_2_O_2_ Eq/L)/TAS (*μ*mol Trolox Eq/L) × 100 [21].

### 2.7. Histological and Ultrastructural Preparations

Small pieces of the pancreas were immediately fixed in aqueous Bouin's solution for 24 h. Paraffin-embedded sections (5 *μ*m thickness) were stained with hematoxylin and eosin [22], microscopically examined, and photomicrographs were made as required. As for the electron microscopic preparations as described by Dykstra et al. [23], freshly excised pancreatic tissue samples were cut into very small pieces and fixed in 2.5% glutaraldehyde for 4 h and 2% paraformaldehyde in 0.1 mol cacodylate buffer (pH: 7.4). The samples were postfixed in buffered solution of 1% osmium tetroxide at 4°C for 1-5 h. This was followed by dehydration in ascending grades of ethyl alcohol, clearing in propylene oxide for two changes, 5 min each, and embedded in EPON epoxy resin. Semithin sections of 1 *μ*m thickness were stained with toluidine blue and investigated under a bright field light microscope. Ultrathin sections were cut, mounted on form var-coated grids, and stained with uranyl acetate and lead citrate [24]. Sections were examined and photographed on a Jeol transmission electron microscope (JEOL Inc., Peabody, MA, USA) in the Regional Center for Mycology and Biotechnology (RCMB), Al-Azhar University, Egypt.

**Figure 1:**
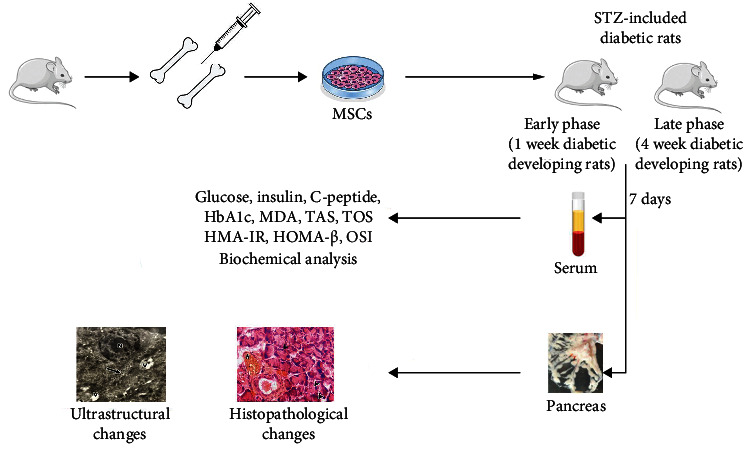
The isolation of MSC and the schematic representation of the study design.

Figure 1 displays a visual summary of the followed methodology and main findings.

### 2.8. Statistical Analysis

All the biochemical data were expressed as mean ± SEM (6 rats/group). The statistical variations between the treatments were evaluated by one-way analysis of variance (ANOVA) using the SPSS/20.0 software followed by Tukey post hoc test. A *p* value < 0.05 was considered statistically significant.

## 3. Results

### 3.1. Biochemical Analysis

**Figure 2:**
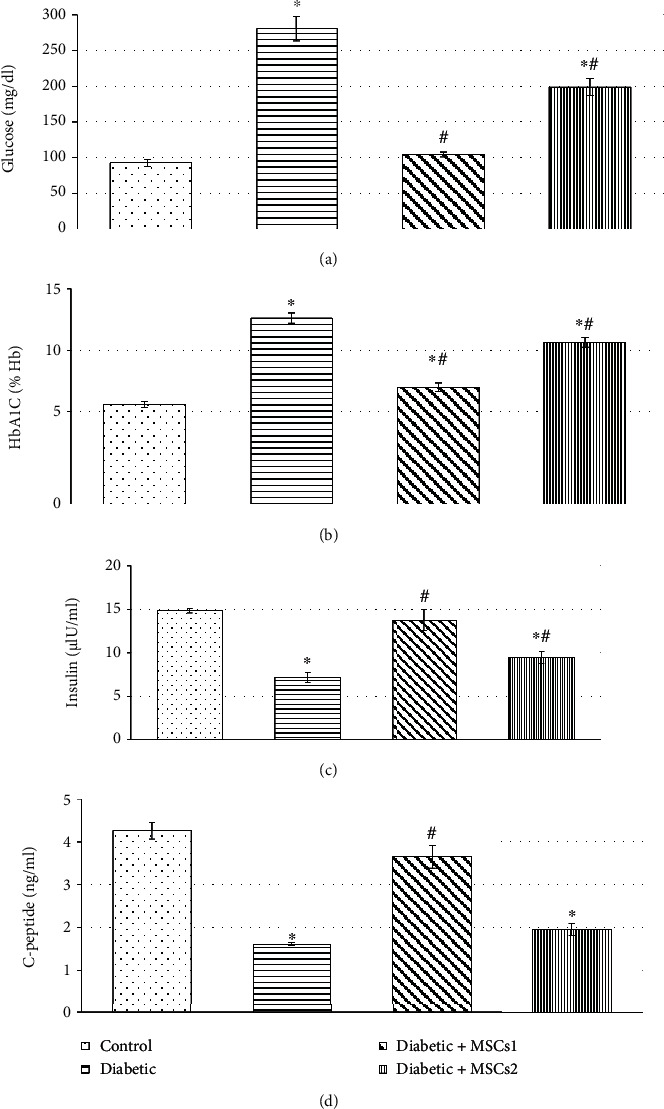
Effect of mesenchymal stem cell transplantation on serum levels of (a) glucose, (b) HbA1c, (c) insulin, and (d) C-peptide of the control and experimental groups of developing diabetic rats. Values are expressed as mean ± SEM (*n* = 6). Comparisons are ^∗^*p* ≤ 0.05 significantly different from the control group and ^#^*p* ≤ 0.05 significantly different from the diabetic group.

Serum levels of glucose, HbA1c, insulin, and C-peptide were assessed to monitor the glycemic state of the control and experimental groups of developing rats. The data in Figure 2 show incredible increase (*p* < 0.05) in serum glucose and HbA1c levels accompanied by marked decline in levels of insulin and C-peptide in the STZ-induced diabetic group as compared with the control group. Meanwhile, the treatment of the diabetic developing rats with a single dose of MSCs during the early phase (diabetic+MSCs1 group) alleviated all the measured glycemic state indices. However, the treatment of diabetic developing rats with a single dose of MSCs during the late phase (diabetic+MSCs2 group) improved the values of these markers relative to STZ-induced diabetic developing rats but these values were still significantly different (*p* ≤ 0.05) compared to the control animals.

**Figure 3:**
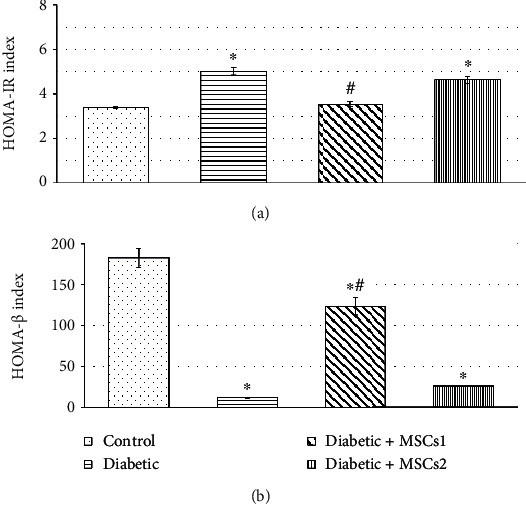
(a) Homeostatic model assessment of insulin resistance (HOMA-IR) and (b) homeostatic model assessment of pancreatic *β* cell function (HOMA-*β*) of the control and experimental groups of developing diabetic rats. Values are expressed as mean ± SEM (*n* = 6). Comparisons are ^∗^*p* ≤ 0.05 significantly different from the control group and ^#^*p* ≤ 0.05 significantly different from the diabetic group.

Figure 3 depicts the impact of MSC administration to developing diabetic rats on insulin sensitivity and pancreatic *β* cell function using two simple mathematical indices (HOMA-IR and HOMA-*β*). Under the current experimental conditions, STZ-induced diabetic developing rats showed higher insulin resistance (HOMA-IR values 5.01 ± 0.16 vs. 3.39 ± 0.04, *p* ≤ 0.05) and lower pancreatic *β* cell function (HOMA-*β* values 12.12 ± 0.55 vs. 182.81 ± 11.64, *p* ≤ 0.05). The supplementation of the diabetic developing rats with MSCs during the late phase caused slight modulation of these parameters; however, they are still significantly different (*p* ≤ 0.05) relative to the control values. On the other hand, the treatment of diabetic developing rats with MSCs during the early phase returned the values of insulin resistance to be very close to those of the control animals (HOMA-IR values 3.54 ± 0.11 vs. 3.39 ± 0.04 ± 0.04, *p* ≥ 0.05) and improved the pancreatic *β* cell function, but the values of HOMA-*β* were still significantly different (*p* ≤ 0.05) from the control ones.

**Figure 4:**
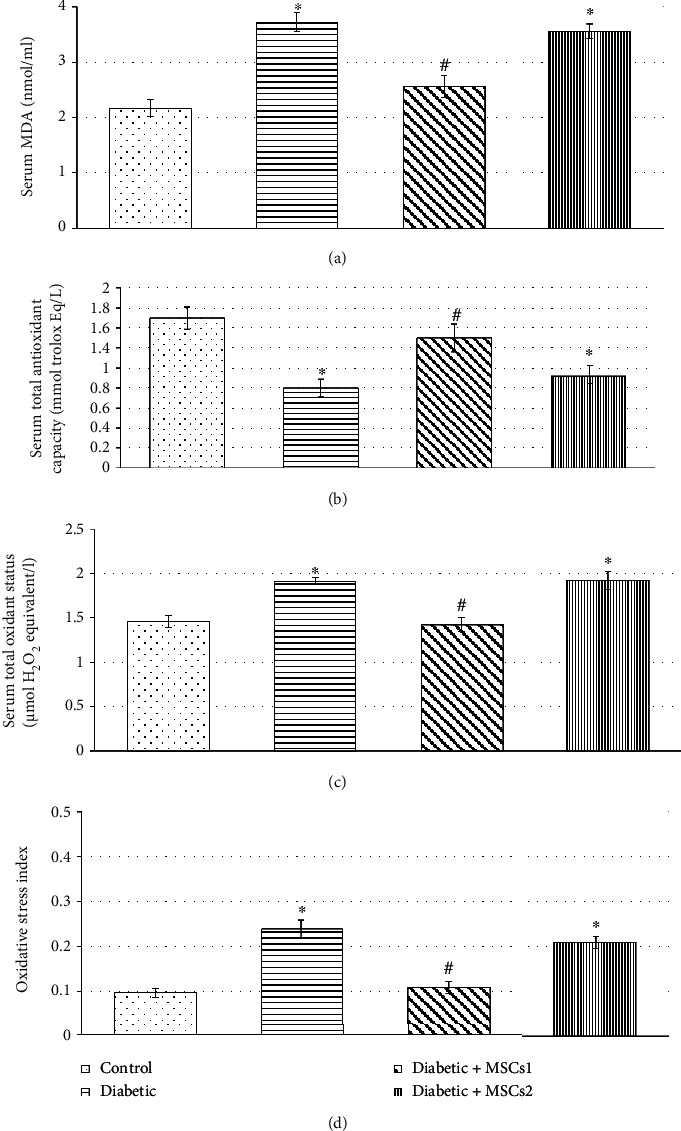
Effect of mesenchymal stem cell transplantation on serum levels of oxidative stress parameters: (a) malondialdehyde (MDA), (b) total antioxidant status (TAS), (c) total oxidant status (TOS), and (d) oxidative stress index (OSI) of the control and experimental groups of developing rats. Values are expressed as mean ± SEM (*n* = 6). Comparisons are ^∗^*p* ≤ 0.05 significantly different from the control group and ^#^*p* ≤ 0.05 significantly different from the diabetic group.

To evaluate the potential effects of intravenous injection of MSC transplantation on oxidative stress indices, the levels of MDA, TAS, TOS, and OSI were determined in sera of the control and experimental groups of developing rats. The obtained results (Figure 4) demonstrated that STZ-induced diabetic developing rats were subjected to oxidative stress which was confirmed by significant reduction of TAS accompanied by marked elevation in levels of MDA, TOS, and OSI compared with the control group. Slight modulation in the values of these oxidative stress indices was recorded in diabetic rats treated with MSCs during the late phase, but these markers are still significantly different (*p* ≤ 0.05) when compared with the corresponding controls. Meanwhile, the treatment of the diabetic developing rats with MSCs during the early phase evoked significant alleviation (*p* ≤ 0.05) in all the measured oxidative stress indices.

### 3.2. Histological and Histopathological Observations

**Figure 5:**
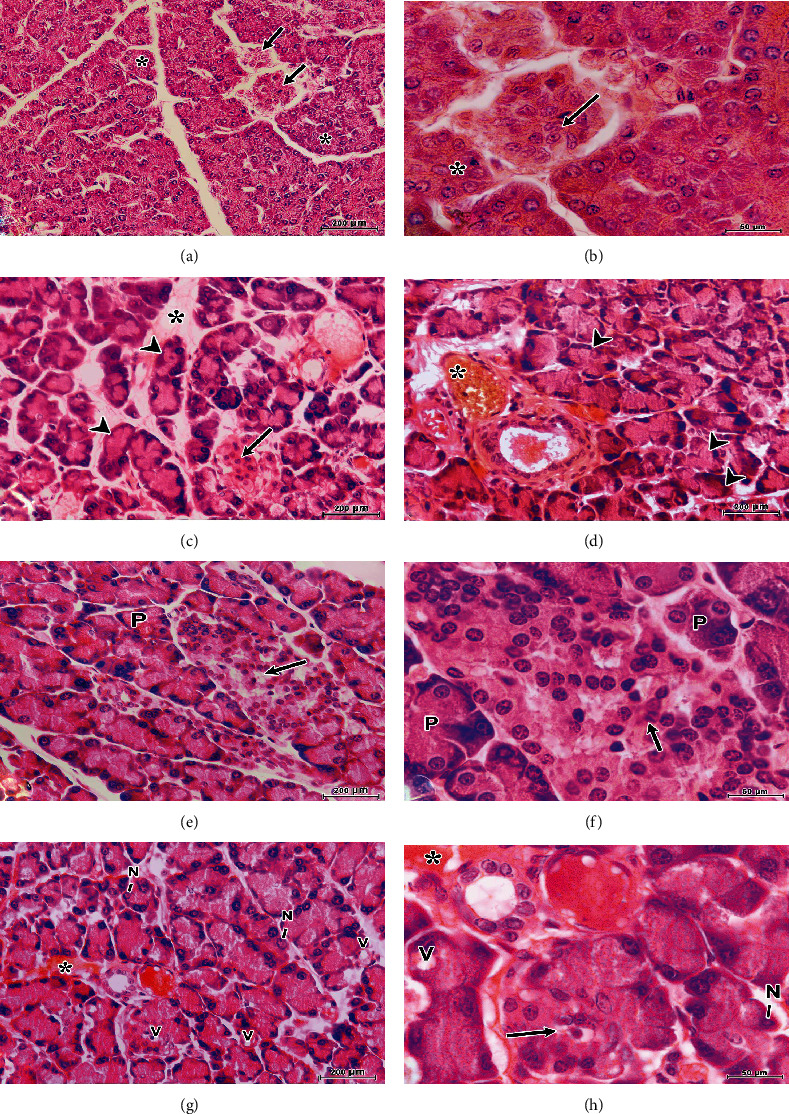
(a, b) Photomicrographs of sections of the pancreas of a control developing rat illustrate closely packed lobules of normal pancreatic acini (∗) and endocrine islets (arrow). The acini are formed of pyramidal cells with basal nuclei and apical acidophilic cytoplasm. (c, d) Photomicrographs of sections of the pancreas of a diabetic developing rat show severe reduction and shrinkage of islets of Langerhans (arrow) with degeneration and necrosis of the acini components (arrowheads) and extravasations of blood from the damaged blood capillaries forming hemorrhagic appearance (∗). (e, f) Photomicrographs of sections of the pancreas of a diabetic developing rat treated with mesenchymal stem cells during early phase (diabetic+MSCs1 group) show normal pancreatic acini (P) and some acinar cells with cytoplasmic degeneration. Note the increase in density (arrow) and size of islet in this group. (g, h) Photomicrographs of sections of the pancreas of a diabetic developing rats, treated with mesenchymal stem cells during late phase (diabetic+MSCs2 group), show no significant recovery. The focal acinar damage represented by cytoplasmic vacuolation (V) and the nuclei (N) exhibited karyolysis of some acinar cells with shrinkage of islets of Langerhans (arrow).

The pancreas of a control developing rat is covered by a thin capsule of connective tissue that sends septa into it, separating the pancreatic lobules. The acini are surrounded by a basal lamina supported by a delicate sheath of reticular fibers. It has a rich capillary network. The pancreas is a mixed exocrine and endocrine gland. The exocrine portion is composed of the acinar cells which are pyramidal in shape with basal nuclei and possess granules at their apex. The intercalated duct penetrates partially into the acini forming centroacinar cells that constitute the intra-acinar portion of the intercalated duct. The endocrine portion of the pancreas is called the islets of Langerhans which dispersed randomly throughout the exocrine portion of the pancreas. The numbers of endocrine cells vary in the islets. Each islet is surrounded by a fine capsule of reticular fibers and consists of polygonal or rounded cells stain by hematoxylin and eosin lighter than (or not heavier than) pancreatic acinar cells and arranged in cords which are separated by sinusoid (Figures 5(a) and 5(b)). There are three different types of cells, but they are hardly differentiated by light microscope.

The histological examination of sections of the pancreas of STZ-induced diabetic developing rats illustrated pathological changes of both exocrine and endocrine parts of the pancreas. The pancreatic acini revealed focal acinar damage ranged from cytoplasmic vacuolation and pyknotic nuclei to acinar necrosis of some acinar cells with extravasations of blood from the damaged blood capillaries forming hemorrhagic appearance (Figures 5(c) and 5(d)). The endocrine part showed extensive damage of the islets of Langerhans which appeared to be irregular in shape with reduction of their size.

The histological structure of the pancreatic tissues of most rats of the diabetic+MSCs1 group displayed normal built-up both in acinar cells and islets of Langerhans except the presence of few vacuoles between the cells of some islets of Langerhans (Figures 5(e) and 5(f)).

Although the pancreatic tissue of some rats in the diabetic+MSCs2 group revealed mild recovery, but most acinar cells exhibited cellular vacuolation with karyolysed nuclei. Damaged blood vessels with extensive edema are also observed (Figure 5(g)), and the reduction of size of islets of Langerhans is also noted (Figure 5(h)).

### 3.3. Ultrastructural Observations

**Figure 6:**
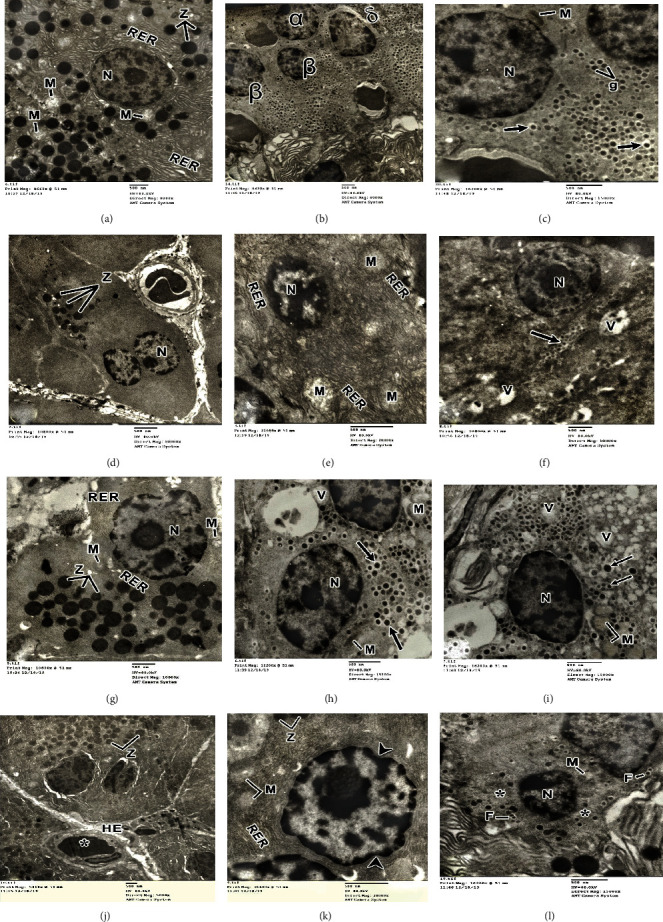
Electron micrographs of a pancreatic acinar cell of control developing rat demonstrating (a) zymogen (Z) granules, arrays of rough endoplasmic reticulum (RER), and mitochondria (M) with basally located nucleus (N); (b) parts of *β*, *α*, and *δ* cells; (c) parts of two *β* cells with spherical-shaped nuclei (N). *β* granules (g) have dark central core surrounded by an electron lucent halo (arrows). Electron micrographs of pancreas acinar cells of diabetic developing rat showing (d) decrease of the zymogen (Z) granules; (e) shrinkage and pyknotic nucleus (N), vacuolized mitochondria (M), and extensive fragmented arrays of rough endoplasmic reticulum (RER); (f) *β* cell in Langerhans islet of the pancreas displaying cytoplasmic vacuolation (V), obvious decrease of secretory granules (arrow), and pyknotic nucleus (N). Electron micrograph of pancreatic acinar cells of a diabetic developing rat treated with mesenchymal stem cells during early period (diabetic+MSCs1 group) illustrating (g) the acinar cells appeared nearly similar to the normal form with basally located normal nuclei (N) and normal aggregation of zymogen (Z) granules with few degenerated mitochondria (M) and arrays of rough endoplasmic reticulum (RER). (h) Aggregation of *β* cells which appeared nearly like the control group, euchromatic oval nucleus (N); few vacuoles in *β* cells and increase of the secretory granules (arrows) surrounded by electron lucent halo; (i) oval nucleus (N); increase in the secretory granules (arrows) with some vacuolized mitochondria (M). Few vacuoles are also seen (V). Electron micrographs of (j, k) pancreatic acinar cells and (l) *β* cell in Langerhans islet of a diabetic developing rat treated with mesenchymal stem cells in a late period (diabetic+MSCs2 group) showing (a) hemorrhage edema (HE) among the acini with congestion in the blood vessels (∗). (b) Damaged mitochondria (M); dilated rough endoplasmic reticulum (RER), zymogenic granules (Z), and an irregular nuclear envelope (arrowheads). (c) Vacuolized mitochondria (M) lost most their cristae and a scarcity of *β* granules in some *β* cells (∗). Fusion of some granules (F) is also seen. The nucleus (N) of islet *β* cell appeared with an irregular shape.

Electron microscopic examination of the pancreas of the control developing rats revealed the fine structure of the acinar cells and islets of Langerhans. The cytoplasm of acinar cells contains numerous arrays of well-developed cisternae of rough endoplasmic reticula, mitochondria, and numerous electron dense secretory granules of variable sizes in the apical part (Figure 6(a)). The nuclei of these cells are basally located and spherical in shape, ensheathed by a nuclear envelope and possessing nucleoli, peripheral dense heterochromatin, and homogenous euchromatin material. The islets of Langerhans of the pancreas of the control developing rats are formed mainly of *β* cells. Their cytoplasm contains numerous electron dense secretory granules surrounded by wide lucent halo and rounded euchromatic nucleus (Figures 6(b) and 6(c)).

Electron microscopic examination of the pancreas of the diabetic group showed marked changes in pancreatic acini represented by dilated and fragmented rough endoplasmic reticula, decrease of secretory granules, vacuolated mitochondria, in addition to shrinkage and pyknotic nuclei. Also, the blood sinusoid was congested by hemolysed blood cells (Figures 6(d) and 6(e)). The *β* cells of the pancreas of diabetic rats showed obvious vacuolation and decrease of secretory granules, fusion of some granules, and pyknotic nuclei (Figure 6(f)).

Marked improvement in pancreatic acini after early treatment with MSCs (diabetic+MSCs1 group) represented by increase in zymogen granules, regular nuclear envelope and flattened rough endoplasmic reticula, and less affected mitochondria (Figure 6(g)). Treatment in the early phase (one-week diabetic developing rats) with MSCs revealed euchromatic nuclei, few vacuoles in *β* cells, and increase of secretory granules compared with the diabetic ones, and the mitochondria appeared nearly normal except that few of them appeared vacuolized (Figures 6(h) and 6(i)).

Electron microscopic examination of the pancreas of diabetic developing rats treated with MSCs (diabetic+MSCs2 group) in late phase (4-week diabetic developing rats) showed conspicuous degeneration in pancreatic acini represented by dilated rough endoplasmic reticula, decrease of secretory granules, degeneration of the mitochondria, in addition to the shrank nucleus which was surrounded by an irregular nuclear envelope. Congested blood sinusoid accompanied with hemolysed blood cells was also seen (Figures 6(j) and 6(k)). *β* cells showed obvious cytoplasmic vacuolation, severe degenerated mitochondria, decrease of secretory granules with fusion of some granules, and pyknotic nuclei (Figure 6(l)).

## 4. Discussion

DM has been ranked as the fifth leading cause of death in the world [25]. The effective role of MSCs in the treatment of T1DM has been reported by several researchers. This is supported with the capacity of MSCs to differentiate into insulin-producing cells that can be used to replace damaged pancreatic *β* cells [26, 27]. In this context, the current investigation was designed to shed light on the potential of MSCs in ameliorating hyperglycemia in STZ-induced diabetic developing male rats, following biochemical, histological, and ultrastructural approaches.

In the present study, T1DM induction was confirmed by the obtained significant increase in serum levels of glucose and HbA1c which was accompanied by marked decline in the levels of insulin and C-peptide in sera of STZ-treated developing rats compared with the control values. Furthermore, STZ injection has resulted in marked elevation in insulin resistance indicated by an increase in HOMA-IR, with a significant decline in *β* cell function indicated by a decrease in HOMA-*β*. These findings are consistent with other studies [28, 29] showing that STZ administration induced progressive *β* cell dysfunction in rats.

STZ is a cytotoxic glucose analogue that penetrates *β* cells via glucose 2 transporter in the plasma membrane [30]. Inside *β* cells, biochemical pathways are induced by STZ causing DNA fragmentation and cell death: (i) DNA methylation through production of carbonium ions which activate the nuclear enzyme poly ADP-ribose synthetase and consequently lead to NAD^+^ depletion; (ii) nitric oxide production, (iii) free radicals' generation, and (iv) altering NF-*κ*B-based cell signaling [31, 32].

Serum C-peptide content is a real predictor of any change in the level of insulin since it is cosecreted by pancreatic cells with insulin as a by-product of the enzymatic cleavage of proinsulin to insulin [33]. HOMA-IR has been shown to be a robust method for the surrogate evaluation of insulin resistance [34]. In the current research, the high values obtained for HOMA-IR in diabetic rats are consistent with Rossetti et al. [35] who proved that high glucose levels induce the development of insulin resistance in peripheral tissues due to impairment of insulin secretion and insulin sensitivity. The biochemical basis for hyperglycemia-induced insulin resistance is still unclear. It may be attributed to modifications in the structure of insulin receptors and the glucose delivery system, resulting in disrupted signal propagation [36].

The observations obtained from the present light and electron microscopical studies clearly demonstrated that the administration of streptozotocin induced various histological and ultrastructural alterations in pancreatic tissues of developing rats. These histopathological changes included degeneration and necrosis of pancreatic acinar cells, with severe damage of the islets of Langerhans. The pancreatic acini revealed focal acinar damage accompanied with extravasations of blood from the damaged blood capillaries (Figures 5(c) and 5(d)). In addition, the pancreatic *β* cells displayed reduction in their size and number as well as marked cellular degeneration represented by cytoplasmic vacuolations and nuclear pyknosis (Figure 6(f)). These results were compatible and confirming the findings of pervious investigators [37–40] in their studies on the pancreas of alloxan-induced diabetic rats. Extravasations of blood from the damaged blood vessels cause the problem that not enough oxygen reaches the pancreatic tissue which leads to tissue degeneration and necrosis [39, 41].

Along the same line, electron microscopic observations of the pancreatic cells of diabetic rats and those of animals treated with MSCs in a late period (4-week diabetic developing rats) exhibited marked alterations in pancreatic acini concentrated in vacuolation and degeneration of the mitochondria, dilation of rough endoplasmic reticula, and minor appearance for the secretory granules (Figures 6(d), 6(e), 6(j), and 6(k)). Also, *β* cells showed reduction in their secretory granules and their nuclei manifested pyknotic appearance (Figures 6(f) and 6(l)). Similar degenerative features were observed in *β* cells of streptozotocin-diabetic adult rats treated with acarbose and *Rumex patientia* L. [42]. It has been reported that mitochondrial dysfunction can lead to cellular damage and apoptosis [43]. In this respect, Bogolepov [44] stated that tissue vacuolation is considered as a structural indication for disturbance in the permeability of the membranes that leads to the transport of water and electrocytes into the cell which may lead to cellular degeneration.

The present investigation showed that infusion of MSCs substantially suppressed damage of *β* cells and enhanced their repair as indicated by the improvement of glucose, insulin, C-peptide, insulin resistance, and *β* cell function. These outcomes are in concurrence with those obtained by Mansor et al. [45] and Hussien et al. [46] who demonstrated that MSCs possess tissue repair and/or cytoprotective potential due to their preferential homing property to damaged pancreatic tissues with significant islet reconstruction. In addition, treatment with a single dose of MSCs has greatly improved insulin sensitivity in diabetic developing male rats. The obtained improvements were most prominent during the early phase (performed one week after induction of diabetes) as compared with the late phase (performed 4 weeks after induction of diabetes). The aforementioned results are confirmed through the histological and ultrastructural observations on diabetic developing rats treated with MSCs in the early period (diabetic+MSCs1 group) which displayed restoration of normal cellular population of the acinar cells and islet of Langerhans (Figures 5(e), 5(f), and 6(g)–6(i)).

Repair of the acinar cells may be due to the rapid differentiation of MSCs into normal functional acinar cells since the presence of immature cells with damage cells help in tissue and cell regeneration [47]. In this respect, several authors suggested the following modes for regeneration. First, the new acinar cells were proliferated from preexisting acinar cells. Second, the degranulated and duct-like acinar cells redifferentiate to a normal acinar state [48–50].

Considering the mechanisms of MSC interactions with the immune response, MSCs possess a wide range of immunoregulatory capabilities that result in immunosuppression of numerous effector functions through interacting with immune cells in both the innate and adaptive immune systems, including B cells, T cells, dendritic cells, natural killer cells, neutrophil, and macrophages [51]. MSCs also promote metabolism by secreting a wide range of chemokines, growth factors, and cytokines, as well as producing a vast array of secretomes and proteomes. These bioactive molecules are involved in immunomodulatory actions, mediating hematopoietic stem cell engraftment, MSC differentiation, and controlling angiogenesis and apoptosis [52, 53].

It has been well documented that MSCs have arisen as helpful means for the treatment of many diseases due to their ability to transdifferentiate in spite of their properties as self-renewal and immunosuppressive potential. In 2013, Wei et al. [54], Dimarino et al. [9], and Patel et al. [10] reported that MSCs could exert their actions through paracrine mechanisms and lead to cellular repairing from damage. Different reports on adult experimental mammals had revealed that there is no evidence for *β* cell regeneration following chemical ablation [55, 56]. Meanwhile, in young rodents, the rate of *β* cell proliferation is quite high and rapidly decreases with age [57–59].

In the current investigation, it was noticed that in the pancreas of developing rats of the third group (diabetic+MSCs1; one-week diabetic rats), the most affected cells were *β* cells than *δ* and *α* cells. This may reveal that a single intravenous injection of MSCs into developing rats at an early age has resulted in the emergence of *β* cells from *δ* and *α* cells. So, early treatment of diabetic rats with MSCs can play an important role in the restriction of type 1 diabetes at an early age. In this regard, Thorel et al. [60] and Chera et al. [61] reported that extreme loss of *β* cell accelerates the conversion of pancreatic *δ* and *α* cells into *β* cells. On the other hand, the extent of such improvement and recovery appears to be reduced greatly in the pancreas of animals of the fourth group (diabetic+MSCs2, i.e., 4-week diabetic developing rats) which means that as the rats became older, the chance for regeneration of acinar cells and *β* cells became insignificant, and this agrees with the explanation reported by Kassem et al. [62], Meier et al. [63], Köhler et al. [64], and Gregg et al. [65] who stated that proliferation of *β* cells in humans occurs only during early childhood.

Finally, as a consequence of our research, we advocate expanding stem cell treatment, particularly in cases of diabetes in children, based on ethical precautions in stem cell sources and in accordance with religion and law. The next step might be to experiment with different stem cell sources and dosages to see how effective they are in curing patients.

## 5. Conclusions

Based on the findings of this study, we can conclude that more MSC therapy at the early stage of STZ-induced diabetic rats and at a young age increases the chance of recovery compared to injury at a later stage and at an older age, and these return to the capacity of cells in the first state in blood glycemic homeostasis restoration and control.

## Data Availability

The data presented in this study are available on request from the corresponding author.
